# Use of monitoring data to improve implementation of a home fortification program in Bihar, India

**DOI:** 10.1111/mcn.12753

**Published:** 2018-12-13

**Authors:** Rukshan Mehta, Reynaldo Martorell, Indrajit Chaudhuri, Amy Webb Girard, Usha Ramakrishnan, Pankaj Verma, Priya Kekre, Sridhar Srikantiah, Melissa F. Young

**Affiliations:** ^1^ Doctoral Program in Nutrition and Health Sciences, Laney Graduate School Emory University Atlanta Georgia; ^2^ The Hubert Department of Global Health, Rollins School of Public Health Emory University Atlanta Georgia; ^3^ CARE India Patna India

**Keywords:** anaemia, Bihar, MNPs, monitoring, process evaluation

## Abstract

This paper describes the use of program‐monitoring data to track program performance and inform activities. Monitoring data were collected as part of an effectiveness trial of multiple micronutrient powders (MNPs) for children 6–18 months in Bihar, India. Communities (*n* = 70; reaching over 10,000 children) were randomized to receive either counselling on infant and young child feeding or both counselling and MNPs. Government frontline health workers (FLWs) implemented and monitored program activities with support from CARE India and university partners. Monitoring data were collected over the duration of the entire program to assess program impact pathways using various checklists, which captured information about (a) attendance and training of FLWs at health subcentre meetings, (b) distribution of MNPs, (c) receipt and use of MNPs at the household level, and (d) midline mixed methods survey. At the beginning of the program, 72% of households reported receiving and 53% reported currently consuming MNPs. These numbers fell to 40% and 43% at midline, respectively. The main barrier to use by household was a lack of MNPs, due in part to infrequent FLW distribution. However, FLWs rarely reported MNP shortages at Anganwadi centres. Side effects also emerged as a barrier and were addressed through revised recommendations for MNP use. Qualitative data indicated high community acceptance of MNPs and a good understanding of the program by FLWs. The use of real‐time program data allowed for recognition of key program issues and decision‐making to enhance program implementation.

**Key messages**

- Program impact pathways are a theory‐driven tool for real‐time monitoring of large‐scale global health pilots and provide essential information on supply and demand side factors, to allow for course correction during implementation.
- Multiple levels of engagement (systems, stakeholder, and community) are necessary for MNP program to be delivered effectively to intended beneficiaries.
- Effective counselling is essential for increased adherence to child feeding recommendations and use of MNPs.

## 1 INTRODUCTION

The state of child anaemia in Bihar, India, is alarming. In 2016, 63.5% of children 6–59 months were anaemic (haemoglobin < 11 g dl^−1^; NFHS‐4, 2016). Micronutrient deficiencies in the first 1,000 days of a child's life can have long‐term impacts on health, future schooling, and work productivity with implications for community and national economic development (Bhandari et al., 2004; Guldan et al., 2000; Zaman, Ashraf, & Martines, 2008). Home fortification with multiple micronutrient powders (MNPs) is a cost‐effective strategy to reduce anaemia, particularly where complementary foods are not adequate to provide those micronutrients essential for growth and development (De‐Regil, Jefferds, & Peña‐Rosas, 2012; Oliveira, Sampaio, Muniz, Cardoso,, & Group., 2016; Santos et al., 2001).

Extensive research documents the efficacy of MNPs, but there exists a dearth of context‐specific evidence on the effectiveness of MNPs for child anaemia reduction both globally and within India (Matias et al., 2017; World Health Organization [WHO], 2011; Yousafzai, Rasheed, Rizvi, Armstrong, & Bhutta, 2014). Our program aimed to test the effectiveness of MNPs to reduce child anaemia in one district of Bihar, India. Recognizing that counselling on child feeding practices is also an important strategy for improving child growth (Home Fortification Technical Advisory Group [HF‐TAG], 2015; Loechl et al., 2009; Robert et al., 2006), we combined home fortification using MNPs with improved counselling on infant and young child feeding (IYCF) practices, as recommended by the HF‐TAG. To ensure sustainability, we implemented the program using existing government health and Integrated Child Development Scheme (ICDS) functionaries.

Few studies have identified context‐dependent bottlenecks, examples of which include supply chain inadequacies, transportation challenges, underestimation during stock and budget forecasting and ensuing delays with payments to manufacturers, poor adherence to dosing requirements, side effects and safety concerns, use of MNPs for nontarget children within the household, and sale of MNP products by the household (Loechl et al., 2009; M. Nguyen et al., 2016; Suchdev et al., 2010; Suchdev et al., 2012). There continues to remain a need for program to systematically study implementation processes (Avula et al., 2013). Additionally, the identification of implementation gaps and demand side barriers is crucial to ensure accurate translation of findings and interpret results related to fidelity of intervention delivery (Habicht & Pelto, 2011; White, 2013). Program impact pathways (PIPs) enable theory‐driven process evaluation, which spans the spectrum from supply to demand and allow for visualization of a program uptake and impact (WHO & UNICEF, 2008). This approach facilitates the examination of contextual factors that influence effectiveness (Avula et al., 2013). Additionally, this framework informs ongoing implementation and guides measures for course correction and integration of needed complementary interventions (Avula et al., 2013). This manuscript describes how we leveraged PIPs to assess the implementation of an MNP effectiveness trial in Bihar, India.

## 2 METHODS

### 2.1 Context and background

CARE India in collaboration with Emory University initiated this home fortification cluster‐randomized controlled trial as part of the broader Integrated Family Health Initiative project. The Integrated Family Health Initiative project, implemented by CARE India in partnership with the Bihar government, aimed to increase universal coverage and quality of life‐saving interventions and improve the health and survival of women, newborns, and children during the first 1,000 days from conception to 2 years of age in Bihar (Smith, Rangarajan, Borkum, & Dandona, 2011).

The home fortification with MNPs program was conducted in two phases, the first involving several rounds of formative research to contextualize the MNPs, IYCF messaging, and study acceptability of the delivery platform (Kekre et al., 2015; Young et al., 2013; Young et al., 2015). Phase two, described here, lasted 12 months and was conducted in 70 communities, designated as the catchments of health subcentres (HSCs). Each HSC has between 5 and 12 Anganwadi centres (AWCs) within its catchment area, and each AWC serves a population of approximately 1,000 people. We chose four blocks for implementation based on distance from the district headquarters. A list of all HSCs in the four selected blocks was generated, and those with political instability or in flood prone areas were excluded from randomization. The remaining 135 HSCs were randomized to generate a list of 35 intervention and 35 control HSCs using a random number generator‐based simple randomization method. Intervention and control HSCs were distributed across all four blocks; hence, risk of contamination or spillover due to geographic proximity could not be completely mitigated. We however monitored for such risks on an ongoing basis throughout the course of the program.

The program was implemented by government frontline workers (FLWs), including (a) Accredited Social Health Activists (ASHAs) and (b) Anganwadi workers (AWWs), who were trained and managed at the level of the HSC, under the supervision of auxiliary nurse midwives (ANMs) and lady supervisors. AWWs are permanent paid employees who receive monthly salaries, whereas ASHAs receive incentives based on completion of tasks, which include counselling mothers on nutrition, pregnancy care, and supporting women to engage in institutional deliveries. Home visits are a routine component of the job descriptions of both, with an average of 50–60 households per month per worker, depending on the number of pregnant, lactating women and children under 2 years of age per community (Khandelwal, Dayal, Bhalla, & Paul, 2014). CARE Innovation Coordinators and HSC mentors, henceforth referred to as program team/staff, provided overall coordination support, were responsible for collection of monthly household and Anganwadi level monitoring data, and provided oversight at the block and HSC levels throughout the study.

MNPs were procured from DSM Nutritional Products in Mumbai, India, and transported to West Champaran district of Bihar, where the study was conducted. Four allotments of MNPs were sent to each intervention AWC from the district headquarters using CARE couriers. We used a cluster‐randomized control design to deliver either MNPs and IYCF counselling to 35 intervention communities or IYCF counselling alone to 35 control communities. FLWs were advised to deliver one box of MNPs to the households of children 6–18 months of age on a monthly basis during routine home visits. Each box of MNPs contained 30 sachets of powders, one sachet to be used per day per child. Counselling simulations were conducted during trainings with FLWs in both groups and utilized role‐playing to demonstrate best practices for household interaction during home visits for MNP and IYCF counselling delivery. Intervention communities received associated instructional materials and behavior change communication (BCC) pamphlets on how to use the powders and recommended IYCF and hygiene‐related practices, with their first delivery of MNPs. Instructional materials for MNP use were presented in graphical form in both the pamphlets and on the box containing MNPs (refer to Supporting Information for pamphlets). In control communities, FLWs were trained to provide counselling and distribute IYCF pamphlets during monthly home visits.

### 2.2 The PIPs

**Figure 1:**
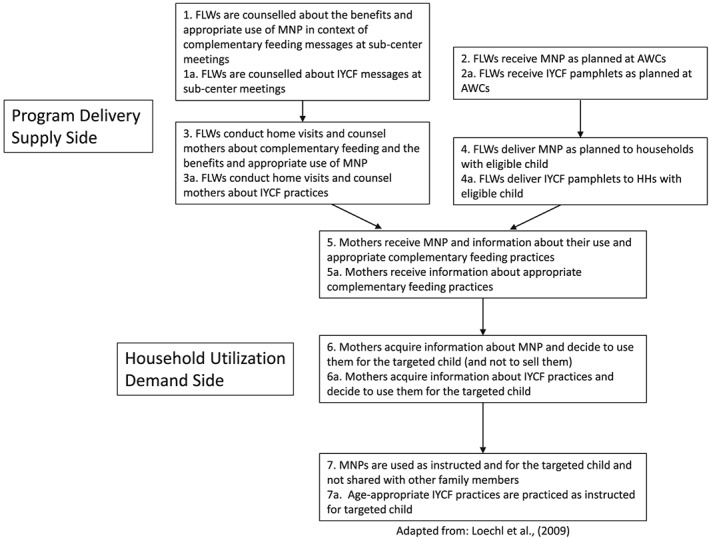
Use of program impact pathways to monitor implementation in intervention and control groups

Program impact pathway (PIP) development is an iterative process and involves the documentation of hypothesized linkages between program activities and their expected immediate, intermediate, and final outcomes (Kim, Habicht, Menon, & Stoltzfus, 2011). We developed PIPs in consultation with CARE India, the implementing partner, and through a detailed analyses of intervention operational materials, technical specifications of MNPs (HF‐TAG, 2015; UNICEF & CDC, 2013), IYCF recommendations (UNICEF, 2011), and other related programmatic areas. The PIP in turn dictated our choice of tools and methods used to gather and analyse data (Figure 1 adapted from Loechl et al., 2009), and reflects aspects related to program implementation (supply) and uptake (demand). An important a priori part of our program design included the handover of all monitoring activities to the government after Month 4. Operationally, this meant scale back of program team involvement in monthly HSC meetings and handover of checklist completion to ANMs.

All data collected from checklists were reviewed by the CARE and Emory teams on a monthly basis and shared with the field program team for dissemination to FLWs. Major decisions with respect to course correction were made primarily following midline data analysis, collaboratively with inputs from CARE, Emory, and government staff.

### 2.3 Data collection

We used a variety of checklists at the HSC, Anganwadi, and household levels, throughout the program at varied frequencies to monitor program rollout, assess the level of program implementation, and evaluate PIPs. At midline, we collected questionnaire‐based data from households and FLWs. Qualitative in‐depth interviews (IDIs) were conducted with caregivers and focus group discussions (FGDs) with FLWs at midline. Table 1 describes data collection protocols in further detail.

**Table 1:** Program impact pathway‐driven process assessment

Method of assessment	Key questions	Frequency and total sample
Supply side checklists		
HSC checklist (completed by ANMs)	Whether trainings were conducted (content of trainings), attendance of FLWs Supply status of MNPs and IYCF pamphlets Whether MNP were delivered timely and in appropriate amounts (adequate recording of MNP delivery—use of home visit planner) No. of home visits conducted (frequency/HH), content of meetings (IYCF/MNP)	70 HSC checklists per month for 1 year. 840 total checklists for the program
AWC checklist (completed by AWWs)	Document FLW fidelity (proper and routine implementation) to the program in intervention HSCs only Assess supply and stock of MNPs at the AWCs Knowledge of FLWs about MNP distribution and use	0 checklists per month during program months: 1, 2, 3, 6, 8, 10, 12 70 total checklists for the program
Demand side checklist		
HH checklist (completed by HH)	Whether mothers received information on MNP and IYCF Adequate distribution of MNP to beneficiaries Reported child feeding practices and use of MNP Monitor contamination (distribution of MNPs and intervention BCC materials) in control HSCs	60 checklists in intervention and 30 in control per month during program months: 1, 2, 3, 6, 8, 10, 12 630 total checklists for the program
Mixed methods midline program assessment		
Quantitative	Caregiver HH survey: Socio‐demographics Child morbidity FLW interactions with the family Receipt and use of MNP IYCF counselling and practices FLW survey: Socio‐demographics Training received by FLWs on IYCF including MNPs Interactions with HHs in catchment areas Knowledge about IYCF including MNP related content MNP distribution and interaction with community members Opinions about the program/intervention and motivation to participate Receipt of payment for work duties	840 HHs and 420 FLWs during Month 4 of the program
Qualitative	**IDIs with caregivers/HHs** Examine MNP acceptability and utilization by target population. Perceptions on importance, value and effects of MNP IYCF KAP Feedback on FLW home visits, information dissemination and product distribution (including MNPs and pamphlets) **FGDs with FLWs** Perceptions on impact on HH visits Perceptions on importance of MNP/IYCF and counselling Perceptions regarding their role in MNP/IYCF distribution and motivation to distribute Perceived community utilization Perceptions on trainings and job aids **IDIs with ANMs (auxiliary nurse midwives)** Experiences as the HSC facilitator and manager of the MNP distribution through this platform Perceptions on importance of MNP and IYCF counselling Perceptions on role of FLWs and distribution of product Perceptions on HSC trainings and job aids	20 HH IDIs 2 ANM IDIs 4 FLW FGDs During Month 4 of the program

*Note*. ANMs: auxiliary nurse midwives; AWCs: Anganwadi centres; AWWs: AWW: Anganwadi workers; FLWs: frontline workers; HSC: health subcentre; IDIs: In‐depth interviews; IYCF; infant and young child feeding; KAP: knowledge, attitudes and practices; MNP: micronutrient powder; PIP: program impact pathway.

### 2.4 Monitoring checklists

All checklists were pilot tested prior to implementation and revised continuously during the initial months of rollout in consultation with the program team and FLWs. This ensured that checklists were not excessively burdensome to complete for field workers (< 30 min), and captured key monitoring information.

#### 2.4.1 Anganwadi checklists

During monitoring months (1, 2, 3, 6, 8, 10, and 12), our study team randomly sampled six HSCs, four in intervention and two in control. From each HSC, 10 AWCs were randomly selected for a total of 30. Checklists were completed by our program staff in consultation with AWWs. Issues with the supply chain identified during data collection were reported to the study coordinator, and supplies were replenished through our network of CARE couriers or frontline program staff. Counselling pamphlets with IYCF messaging (in control and intervention communities) and instructions for MNP use (in intervention communities only) were also replenished via our program staff based on information gathered from these checklists.

#### 2.4.2 HSC checklists

HSC checklists (refer to Supporting Information) were completed monthly by our study team and by ANMs across all 70 study HSCs. We trained ANMs to complete these independently with the aim to handover this activity to them once initial program staff involvement was scaled back. The checklists were provided to ANMs at weekly meetings held at block‐level primary health centres and collected at this forum or at HSC meetings by program staff.

#### 2.4.3 Household checklists

In intervention and control communities, program staff completed household monitoring checklists with caregivers and those responsible for the feeding of children aged 6–18 months. Checklists were collected monthly for Months 1 to 3 and then bimonthly for Months 6 to 12. Staff did not complete checklists in Month 4 or 5 as these coincided with the midline. During each monthly monitoring, four intervention HSCs and two control HSCs were selected randomly and checklists completed for 60 intervention and 30 control households. Program staff targeted two AWCs per HSC for household monitoring and randomly selected households for monitoring based on catchments targeted during that month.

#### 2.4.4 Program implementation midline surveys

The midline was conducted in Month 4 to assess program rollout and implementation. We used data from midline to determine the duration of rollout and the timing of program endline. To understand supply and distribution, we assessed FLW reported delivery of MNPs and caregivers reported receipt of MNPs at the household within the last month. To measure uptake, we looked at whether caregivers reported current use of MNPs/child having consumed MNPs in the last month and week.

Low program implementation was defined as <40% of households reporting receipt and uptake of MNPs in the last month, medium as <60%, and high as >60%; these cut‐offs were defined a priori by the team and were based on researcher judgement. At midline, we interviewed 840 caregivers with children 6–18 months of age, and 420 FLWs. All 70 HSCs were sampled; four AWCs were randomly chosen per HSC; four households per intervention Anganwadi and two per control Anganwadi were chosen randomly and interviewed by our program staff based on household lists provided by FLWs from their registers. We also conducted qualitative IDIs with caregivers (*n* = 20) and ANMs (*n* = 2) in addition to FGDs with FLWs (*n* = 4) from one low‐ and one high‐performing intervention HSC. The “high” and “low” performances were decided based on HSC and household level‐monitoring data and in consultation with our program staff and ANMs who supervised FLW attendance at meetings, using indicators of home visits and overall performance. The quantitative survey took 1 month to complete, whereas qualitative data collection was completed over the course of 14 days.

Quantitative analyses were conducted in SPSS; we report percentages for key indicators. The first author and members of the CARE program team, all of whom had prior training and extensive experience with qualitative methods, facilitated qualitative research. Facilitators conducted IDIs and FGDs in Bhojpuri or Hindi depending on the respondent's preference using semistructured guides. IDIs were conducted within the household, and FGDs were conducted at a central location within chosen communities. Qualitative FGDs averaged seven participants. IDIs and FGDs were recorded with participant permission, transcribed as detailed summaries, and then translated to English by a trained Bhojpuri‐English translator. Facilitators and/or note takers also took extensive field notes during FGDs and IDIs that contributed to detailed summaries. Translated detailed summaries were cross‐verified by the first author, in a subset to ensure concordance with recordings. The first author summarized qualitative findings using manual analysis supported by MS Word. We coded detailed summaries (mix of inductive and deductive) and assessed key themes using a thematic analysis approach (Avula et al., 2013; Gale, Health, Cameron, Rashid, & Redwood, 2013; P. H. Nguyen et al., 2014).

### 2.5 Ethical approval

Approval for the study was obtained from the Futures Ethics Board in New Delhi, India, and Emory University's Institutional Review Board. Informed consent was collected from all respondents prior to data collection.

## 3 RESULTS

For the purposes of this process evaluation, we classified all data collected from FLWs (HSC and AWC checklists) as generating information about the supply side and all data collected from households (HH checklists), informing demand side indicators. Monitoring was also carried out in the control group to track spillover receipt/use of MNPs. We did not identify issues with spillover and have therefore reported results from intervention group data only. Results from the midline mixed methods data complemented monthly monitoring results. Table 2 summarizes results regarding facilitators and barriers to program implementation and steps taken to rectify issues arising as part of our data driven design.

**Table 2:** Key issues and resolutions identified using program impact pathways

	Facilitators identified	Issues identified	Resolutions
Program delivery—supply side PIPs (FLW survey, AWC checklists, HSC checklists)			
FLWs are educated about the benefits and appropriate use of MNP in context of complementary feeding messages at subcentre meetings	HSC meetings held regularly with good engagement of ANMs, lady supervisors, and CDPOs.	No. of HSC meetings held declined in Months 4 through 6, as program lost momentum, coinciding with end of program team engagement	Program team reintegration after midline to increase oversight and supportive supervision to FLWs *Systems level engagement* of district level health and ICDS officials to provide supportive supervision and monitor FLWs; this escalation to and engagement of higher levels of the systems was meant to build sustainability and ownership for eventual handover and scale‐up Program team attended monthly meetings to retrain/offer refreshers to FLWs on MNP related topics and IYCF technical content
FLWs receive MNP as planned at AWCs	Timely delivery of MNPs to all AWCs and replenishment of stock in case of shortage.	Stock‐outs of MNPs due to lack of supply in remote AWCs/or those without proper attendance/representation at HSC meetings	Stock replenishment from district central storage to FLWs/AWCs by CARE couriers, based on information gathered from checklists, with staggered distribution of supplies, prioritizing areas with greater need depending on availability of stored stock
FLWs conduct home visits and educate mothers about complementary feeding and the benefits and appropriate use of MNP	FLWs visit all eligible beneficiary HHs monthly and provide adequate counselling on IYCF and MNPs	FLWs not conducting home visits FLWs not providing adequate counselling on MNPs and IYCF practices	Program team provided support to FLWs to encourage home visits and provided support for completion of home visit planners To reduce FLW home visit burden, recommendations revised to have FLWs deliver 3 boxes of MNPs to households during one visit and to also encourage households to pick boxes of MNPs up from the AWCs directly when HH supply is over Supportive supervision and retraining of FLWs to improve quality of counselling provided at HHs Community meetings to convey counselling messages to HHs, supplementing home visits
FLWs deliver MNP as planned to households with eligible child	FLWs enroll children becoming eligible on an ongoing basis, over the duration of the program and provide home visits, MNPs and counselling FLWs provide ongoing follow‐ups to eligible households and help mothers with problem solving around MNPs if needed	FLWs not delivering MNPs to households on a monthly basis	To incentivize home visits, post completion of the program, by FLWs we awarded prizes (household items including cooking utensils and appliances) to 3 top performing FLWs in each block during block level meetings held as part of our *stakeholder engagement* and results dissemination strategy. We also provided MNP logo bags to all FLWs from study communities during this process as a token of appreciation.
Household utilization—demand side PIPs (HH survey and HH checklists)			
Mothers receive MNP and information about their use and appropriate complementary feeding practices	Mothers who receive MNPs, are feeding their children age appropriate complementary foods, following other IYCF recommendations	FLWs not delivering MNPs to households on a monthly basis HHs not following up with FLWs to demand product/not visiting AWCs to replenish HH supply	*Community engagement meetings* held to increase demand generation at the household level HH counselling provided to mothers on initiation and age appropriate IYCF practices, responsive feeding and persistence in feeding children in addition to use of MNPs by FLWs
Mothers acquire knowledge about MNP and decide to use them for the targeted child (and not to sell them)	Mothers who are practicing age appropriate IYCF behaviours, and see benefits of MNPs, use them regularly and as per instruction for target child	Mothers did not use MNPs because they perceived/child did experience side effects or that child did not like the taste Mothers did not use because child was not taking complementary foods Mothers not giving MNP to child because he/she is unwell, not eating	HHs counselled on side effects, including temporary black stool, reduced dosage to control vomiting and spitting out For children 6–9 months who reported side effects and are just starting complementary feeding, reduce dosage of MNPs mixed in food per meal by splitting sachet into 1/3 for each meal during the day Revised recommendations for IYCF during illness and messaging to encourage MNP use
MNP are used as instructed and for the targeted child	Tailored counselling messages received to address child feeding concerns Mothers and families see benefits of feeding children MNPs and continue appropriate use	MNPs used in dry rice, which leads to child spitting them out	Mothers counselled by FLWs and during *community engagement meetings* to add MNPs to food that has mixed wet and dry components (e.g., rice mixed with lentils and vegetables) to increase palatability and reduce detectability of powder in food HHs told not to add MNPs to very hot food (causes dissipation of maltodextrin coating of MNPs, which can causes changes to taste and colour of food)
		Reports of MNP packets being spoiled due to moisture content in storage (MNPs congealed to form hard solid substance)	We informed the manufacturer of issues with some packets of MNPs and additional measures were taken to ensure proper packaging during production, adequate storage of MNPs in spaces without moisture or risk of dampness both during transport and once in central storage at the district level. FLWs were also retrained on proper storage of stock at AWCs We delivered messages through community meetings and FLW trainings to dispose of packets of MNP that had congealed or gone bad

*Note*. ANMs: auxiliary nurse midwives; AWCs: Anganwadi centres; FLWs: frontline workers; HSC: health subcentre; IYCF; infant and young child feeding; MNP: micronutrient powder; PIP: program impact pathway.

### 3.1 Program delivery—Supply side PIPs

#### 3.1.1 Training of FLWs

**Figure 2:**
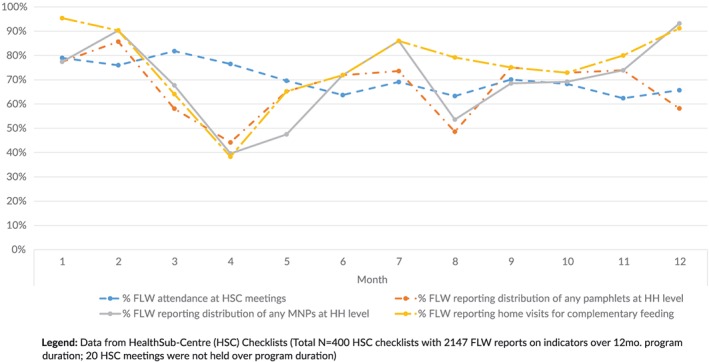
Supply side micronutrient powder (MNP) program monitoring indicators for intervention group. FLW: frontline worker

In our intervention group, HSC meetings were held regularly throughout the course of the study with over 90% of HSCs reporting monthly meetings. In the initial months, CARE program staff helped coordinate meetings in collaboration with government staff. We saw a dip in the number of meetings held in Month 4. An a priori component of the program design required that all HSC meetings be coordinated and checklists be completed by ANMs, during monthly meetings without external support from our program team following handover after Month 3. The dip we see in our key indicators during Month 4 coincided with this planned handover of program leadership to ANMs and lady supervisors (Figure 2).

Overall knowledge of FLWs on MNPs remained high throughout the program and ranged between 87% (Month 1) and 100% (Months 3, 6, 8, 10, and 12, with an overall mean of 97%) of workers surveyed using AWC checklists reporting the correct age for MNP introduction. Between 63% (Month 1) and 100% (Months 2, 6, 10, and 12, with an overall mean of 90%) of workers reported the correct age to stop MNP use, and 100% (all months) of AWWs correctly reported amount for daily consumption over the study period. Additionally, between 86% (Month 4) and 100% (Months 1, 2, 6, 7, and 12 with an overall mean of 97%) of all intervention group FLWs attending monthly HSC meetings reported receiving training on MNPs. We saw the lowest percentage in Month 4 of implementation when program staff activity was scaled back as part of program handover (Figure 2).

#### 3.1.2 Home visits and distribution of MNPs

HSC checklist reports of home visits in the first 2 months of the program were very high (95% and 90% reporting home visits in intervention HSCs and 99% and 98% reporting home visits in control HSCs in Months 1 and 2, respectively), with uneven decline thereafter, as seen in Figure 2. As we reintroduced program staff involvement in coordination of HSC meetings and ramped up supportive supervision from government counterparts in integrated child development scheme (ICDS) and Health, we noted an improvement in home visit patterns (86% at Month 7), followed by stabilization throughout the remaining months of the program (91% at Month 12). The overall data indicate variability in MNP distribution patterns for FLWs over the course of the program, ranging between 40% (Month 4) and 93% (Month 12 with an overall mean of 70%) reporting distribution of MNPs at the household level on HSC checklists. AWC checklist data, which represent a subset of FLW monitoring responses, show that 89% (Month 1) and 100% (Months 2, 6, 8, 10, and 12 with a program mean of 98%) of AWWs surveyed reported distributing MNPs over the duration of the program. There was considerable variability in the reporting of supply shortages by the AWWs in AWC checklists (25% in Month 1, 3% in Month 2, 17% in Month 3, 3% in Month 6, 90% in Month 8, 10% in Month 10, and 20% in Month 12). In the eight month of program rollout, we saw a spike in MNP shortages (90% of AWCs reporting shortages), as this period fell right before round III of MNP distribution in the field, but this situation was rectified immediately, with recovery seen in the indicator during monitoring in Month 10 (10% reporting shortages).

### 3.2 Household uptake—Demand side PIPs

**Figure 3:**
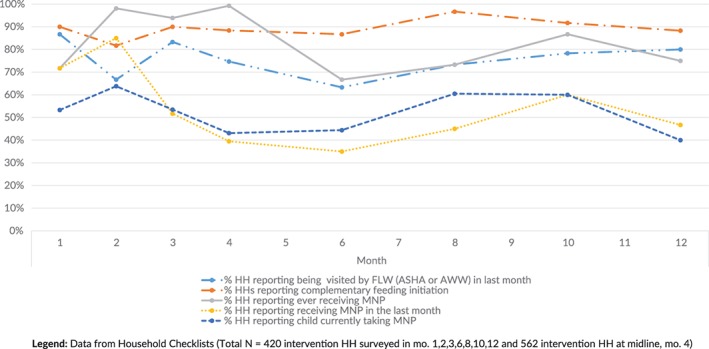
Demand side micronutrient powder (MNP) program monitoring indicators for intervention group. ASHA: Accredited Social Health Activist; AWW: Anganwadi worker; FLW: frontline worker

To help us understand the demand side of program implementation, we collected data on home visit patterns from the perspective of households and uptake of MNP and IYCF practices using household checklists (Figure 3). Our findings show that on average, 54% (35–85%) of households reported receiving MNPs in the last month and 52% (40–63.8%) of households reported their child was currently taking MNPs, over the course of the program.

#### 3.2.1 MNP and IYCF pamphlet receipt

Receipt indicators at the household level show patterns similar to our supply side indicators for the first 3 months of the program. Percentages for households reporting *“ever”‐receiving MNPs* were relatively high (72%, Month 1; 98%, Month 2; and 94%, Month 3) early in the study, dipped in Month 6 (67%), followed by a steady upswing (73%, Month 8; 87%, Month 10; and 75%, Month 12). Additionally, 55% (45–73%) of households on average reported being visited by ASHAs and 57% (38–73%) by AWWs in the last month; 61% (47–78%) of households on average reported receiving IYCF pamphlets over the duration of the program, and 55% (25–64%) reported finding the pamphlets useful and informative.

#### 3.2.2 IYCF practices and MNP‐related knowledge and use

In the intervention group, throughout the course of the study, a mean of 74% (63–87%) of households reported being visited by FLWs in the last month. Approximately 89% (82–97%) of households in intervention group reported correct knowledge about initiation of complementary foods at 6 months. Additionally, 67% (53–89%) of households on average, over the span of the program, reported currently using MNPs if received. Finally, on average, over the course of the program, 34% (11–51%) of eligible children were not consuming MNPs, as observed in household monitoring data.

#### 3.2.3 Side effects

We noted through household and HSC checklists and during routine contact with FLWs that children 6–9 months who had initiated complementary feeding concurrent to the introduction of MNPs more often reported side effects such as “vomiting” or spitting up of MNP fortified food, and blackened stools.

A child between the ages of six to eight eats much less food, compared to that quantity of powder is more. Because of this imbalance in proportion of food and powder, child refuses to eat food. When food is more and powder is less, child does not recognize the presence of the powder in the food. FGD with AWW in low‐performing HSC

We addressed the issue of side effects such as vomiting, through retraining of FLWs. As well, we organized community meetings to support demand generation and to provide additional sources of counselling and support to community members. We modified FLW trainings on feeding recommendations for children experiencing side effects based on strategies, first suggested by FLWs themselves, elucidated through participatory consultations. To this end, all FLWs were advised to counsel mothers to split MNP sachets across 2–3 meals per day. These recommendations were well accepted and helped improve overall uptake.

I ask the mother to take some cooked rice in a clean bowl and also bring pulses curry or vegetable curry or anything which is cooked. Then I mix one third of the powder by tearing the packet and telling her what she has to do, and also say that if you will give like this, the child will not vomit and eat the powder in mixed food. FGD with ASHA in low‐performing HSC

### 3.3 Program implementation midline survey

The program implementation midline was conducted in Month 4 of MNP rollout. Our results show that overall implementation was low. Receipt of MNP in the last month, as reported at the household level, was low (40%), as was consumption (35%), as presented in Table S1. These data were used to help determine the timing of the endline survey and inform other programmatic course corrections.

#### 3.3.1 Acceptability and use of MNPs

Overall, program exposure and acceptance of MNPs, as measured at midline, was high. Approximately 76% of people in the intervention group reported ever having heard of or seen the MNP, and 71% of households reported consuming MNPs in the last week, if received supply in the last month. FLWs and households expressed positive opinions of the MNPs.

People told me that it is good for the health of the child. This will increase strength and weight of the child. So, for the good of the child I am giving this powder regularly to my child. HH IDI with mother in low‐performing HSC

There is no problem sir, child is getting fat and the mind also grows. FGD with ASHA in low‐performing HSC

#### 3.3.2 Barriers to MNP use—Identified at midline

A lack of supply of the powders in the household was identified as the most common reason for not using the powders (21%), implicating bottlenecks in our supply chain. At midline, households also described several additional reasons for not using MNPs: forgot to give MNP (3.4%), side effects (5.9%), poor appetite of child (3.4%), and illness including cough, cold, or fever (3.0%).

#### 3.3.3 Counselling

A lack of adequate counselling on how to use MNPs emerged as a common concern for poor uptake in both the quantitative household survey and qualitative IDIs with households. Some households reported mixing the MNPs in plain rice (without lentils or sauces) and feeding the child, often leading to children spitting out the powders.

Generally, child eats rice when it is mixed with oil, salt and chilli powder. When child is given rice mixed only with powder, she spits it out from her mouth. HH IDI with mother in high‐performing HSC

Additionally, we added messaging to advise use of MNPs in wet foods (rice mixed with lentils and vegetables to ensure it is moist), where the powders are mixed in completely and therefore undetectable to the child.

The issue of side effects and feeding during illness emerged during qualitative work and in routine monitoring.

When child is sick, she does not eat just milk is given. HH IDI with mother in low‐performing HSC

Child resists food when he feels sick. When child resist food we do not force much. HH IDI with mother in low‐performing HSC

We retrained FLWs on counselling mothers with ill children, by emphasizing the importance of responsive feeding with attention to quantity and frequency of feeds during illness.

#### 3.3.4 Delivery platforms

Additional findings from the program implementation midline show that 40% of families reported receiving the powders by visiting the Anganwadis as opposed to home visits. We revised our delivery approach to include pick‐up of MNPs at Anganwadis directly, in addition to home delivery by FLWs.

Many people are not aware of the importance of the powder, for that at initial stage ASHA and Aanganwari worker have to go to each child's home for distribution of the powder. When people start using powder they themselves will collect it from the AWC. IDI with ANM in high‐performing HSC

To alleviate FLW work burden and ensure adequate household stock over the festival season, we amended recommendations to encourage FLWs to deliver three boxes of MNPs to each household at a time. This two‐pronged approach to dissemination of the powders catered to the needs and convenience of individual families and FLWs.

#### 3.3.5 Working relationships and community perceptions of FLWs

The qualitative data showed that in areas where FLWs shared a good working relationship, effort was distributed relatively evenly between both cadres of workers. Approximately 21% of households reported receiving the powders from ASHAs and 35% reported receiving MNPs from AWWs.

The powder is dropped at AWC first, then Aanganwari worker informs the respective ASHA about the powder, and planning for the distribution is done based on the requirement of the households. FGD with ASHAs in high‐performing HSC

When asked, about their overall relationships with the communities they serve, FLWs reported that they had improved through the program:

One positive side of this program when ASHA and AWW are involved in such programs they are earning good will of people. People benefitting from this powder consider them the benefit provider. IDI with ANM in high‐performing HSC

It was not like we have to give this powder to some and not to others. It has to be given to all the eligible children of the area who are of a certain age group. Since the beneficiaries are in almost each household, they get benefit. They think that the Aangawari sister has come with something for their children. And rest of the things are based on categories which create dissatisfaction, that few of them are getting and many are not. AWW FGD in high‐performing HSC

As shown in Table S2, 98% of FLWs at midline thought it was important to provide MNPs to children in their communities, 96% agreed that MNP distribution had improved their overall status in the community, and 96% said they would continue to distribute MNPs in the future.

#### 3.3.6 Remuneration and payment for FLWs

We observed that 92.6% of FLWs reported not receiving full payment of regular salaries during our midline. Additionally, 64.4% of AWWs and 63.8% of ASHAs reported never receiving their routine payment on time.

Kindly tell us Sir, is it possible for a family to survive on remuneration of Rs 3000? All the field work has to be done by us but the government is not thinking of us …. FGD with AWWs in high‐performing HSC

No additional compensation was provided to FLWs for their participation in this program and resultant increase in workload. These issues collectively were an essential bottleneck in our supply chain that were beyond the scope of this program to address.

#### 3.3.7 Systems, stakeholder, and community engagement in implementation

Results from midline data were leveraged to engage all levels of the government system within the district. We worked with the district program officer to improve supervision provided directly to FLWs by community development program officers, lady supervisors, and ANMs. We also increased community demand generation by hosting two rounds of additional community meetings in Months 8–10 and 11–12, to inform and educate beneficiaries about the program, MNP use, and provide information on IYCF, supplementing counselling during home visits by FLWs. We held block level meetings with all FLWs to go over data from midline and ongoing monitoring activities, as part of our stakeholder engagement strategy. We deliberately shared data with them to help boost morale and inform them of the results of their work. At the end of the program (Month 12), we distributed inexpensive household items as prizes, through block level government officials, to three top performing FLWs in each block to recognize their commitment, work ethic, and contribution to the study. In addition, all FLWs were provided with inexpensive program (MNP logo) carry‐bags to recognize their contributions.

## 4 DISCUSSION

PIP analysis was used to help understand the implementation and uptake of the MNP program in Bihar, India. We used our monitoring data to (a) guide MNP field distribution; (b) target systems, stakeholder, and community engagement activities; (c) dictate the overall duration of the program; and (d) track overall program progress.

The mixed methods process evaluation approach informed ongoing decision‐making, allowing us to gather in‐depth information about issues arising in the field, develop responses that could be quickly integrated back into the program, and monitor whether responses adequately addressed issues. For example, an issue discovered during routine monitoring was the management of side effects, for which we revised MNP dosage recommendations.

Routine monitoring also helped us track implementation, by having our field teams conduct surveys regularly, visit more remote study sites during the trial, and address supply chain issues in real time. Ongoing training was an essential component of program implementation and improves quality of care delivered and communication by FLWs as has been reported in prior research (Avula et al., 2013; Bryce, Victora, Habicht, Black, & Scherpbier, 2005). Reinstating our program team to provide refresher trainings and continuously reiterate program‐related content was a key decision taken after looking at monitoring and midline data.

The overall goal of this study was to assess whether program scale‐up would work using existing government structures. Measures to encourage future program sustainability were put in place wherein CARE program staff were never directly involved in delivery of the intervention to households. Our team did retain control of the supply chain from manufacturer to decentralized storage sites (AWC), however due to which we were able to ensure that stock‐outs were not an issue up to this point. Scale‐up of this intervention would require additional inputs from the government to manage these higher rungs of the MNP supply chain from manufacturer to storage facilities. Our program team was also involved at higher levels to boost performance by FLWs.

We recognize the impact of potential Hawthorne effect created by intense monitoring activities such as those carried out during this study and that this may influence indicators more than would be seen if carried out solely by existing government structures (McCambridge, Witton, & Elbourne, 2014). Our learnings reinforce, however, that new program require increased systems involvement from the get‐go, initial investment in inputs for FLW training, and community demand generation to ensure adequate program uptake (MNP utilization and consumption; Mason, Sanders, Musgrove, & Galloway, 2006).

The training of and provision of supportive supervision to FLWs, in addition to their use in the delivery of program such as these, builds capacity and sustainability. A recent review states that FLWs are the most common delivery channel for MNPs identified in the literature to date (Reerink et al., 2017). However, such engagement is complicated by existing work responsibilities (Avula et al., 2013; Nair, Thankappan, Sarma, & Vasan, 2001). FLWs are often overburdened and underpaid, which likely dilutes the quality and consistency of service provision (Glenton et al., 2010; Nair et al., 2001; Standing & Chowdhury, 2008). The role of adequate and timely compensation for frontline implementers is of critical importance to ensure the success of effectiveness program that aim to reach the most underserved populations (Avula et al., 2013). A recent study conducted in Bihar shows promising results for improved teamwork, empowerment, job satisfaction, and equitable service delivery, in addition to higher levels of motivation among FLWs, employing a team‐based goals and incentives model (TBGI) (Grant et al., 2018). Provision of gifts and rewards to some FLWs and not others may be demotivating; however, results from the team based goals and incentives (TBGI) intervention show that public recognition and teamwork can enhance levels of motivations. Other studies have noted that FLWs trade‐off current opportunity costs of low/no paying community health work against future aspirational benefits. Findings show however that continued lack of remuneration and other extrinsic incentives lead to worker dissatisfaction and undermine intrinsic motivations (Kasteng, Settumba, Kallander, Vassall,, & inSCALE Study Group., 2016). Similarly, Strachan et al. (2012) note that failure of program to deliver on expectations of FLWs, centred around receipt of rewards following efforts, can be viewed as a breach of trust, and severely impact overall worker quality and retention (Strachan et al., 2012).

Beyond the delivery of the intervention, several demand side factors should be considered. We were able to use PIPs to examine critical linkages between implementation and uptake (Loechl et al., 2009; Suchdev et al., 2010). Adequate counselling and education on the purpose, use, and benefits of MNPs is essential to ensure acceptability and fidelity at the household level. This includes management of side effects associated with MNPs, use, feeding during illness, and hygiene practices necessary to reduce morbidity. Other authors have noted a reduction in the quantity and frequency of complementary feeds given to children during illness episodes. There remains a need for standardized and consistent counselling messages to support mothers in feeding children during illness episodes (Brown, Stallings, de Kanashiro, de Romana, & Black, 1990; Hoyle, Yunus, & Chen, 1980). Additionally, mothers must have the capability, opportunity, and motivation to engage in behaviour change necessary to ensure adequate IYCF practices (Michie, van Stralen, & West, 2011). Resource limitations particularly in low‐income settings may hamper the adoption and practice of recommended IYCF behaviours both during illness episodes and otherwise.

We found in our routine monitoring and qualitative work that the small quantities of food consumed by children 6–8 months of age, in particular, hampered palatability of MNPs. Other authors have suggested that children who have just started complementary feeding take time to adjust to food intake; simultaneous initiation of MNPs, particularly in smaller volumes of food, may lead to side effects such as vomiting during this period of adaptation to CFs (De‐Regil, Suchdev, Vist, Walleser, & Peña‐Rosas, 2013; Gove, 1997; Suchdev et al., 2011; Suchdev et al., 2012; Suchdev, Peña‐Rosas, & De‐Regil, 2014). Our recommendation, developed in consultation with FLWs, to reduce the quantity of MNPs used per meal, especially for children ≤9 months and those experiencing side effects, by dividing one sachet into three meals per day was well accepted by community members and FLWs. Counselling was provided on hygienic storage of opened packages, away from moisture and light. We acknowledge that this recommendation may increase risk of contamination of packages, if not sealed and stored adequately.

Previous studies have focused on either implementation or uptake (Bonvecchio et al., 2007; Guldan et al., 2000; Santos et al., 2001), but few have taken a holistic approach to understanding program impact across the supply chain (Bhandari et al., 2004; Santos et al., 2001; Zaman et al., 2008) or uptake (Bhandari et al., 2004; Zaman et al., 2008). We noted differences in patterns of implementation (FLW reports of MNP distribution) versus receipt and uptake, in our monitoring data. This highlights the need for triangulation of information that looks at both sides. The utility of this approach is its ability to reduce the influence of social desirability bias on interpretation of data by providing a well‐rounded picture that seeks to validate the needs and opinions of service providers, beneficiaries, and intended users. Additionally, this approach enabled timely action on modifiable factors that, in the case of this program, include efficient delivery and replenishment of stocks correction of improper counselling about MNPs, and modification of delivery modalities to accommodate pick up at Anganwadis and delivery to the household. Non‐modifiable factors such as salaries not received on time by FLWs are also a likely hindrance to the success of new interventions integrated into existing services. Such data provide essential context to our overall findings.

Limitations of our approach include small sample sizes for monthly and bimonthly monitoring activities, and burden associated with routine data collection. We were not able to comprehensively test the knowledge of FLWs after providing trainings, and we did not provide monetary or nonmonetary incentives for their work. Our data are not representative of the population and are based on self‐report. These findings span a short time period (1 year), as opposed to several years, thus highlighting an important evidence gap and need for monitoring of MNP program that have a greater longevity.

In addition to PIPs, other authors have devised a global framework for reporting of context and implementation for pilot studies to be utilized in intervention trials (Luoto, Shekelle, Maglione, Johnsen, & Perry, 2014). The uptake of these criteria alongside PIPs in the development of pilots will add to systematizing methods focused on monitoring of large‐scale community‐based program in low‐ and middle‐income countries. There is a need to balance requirements for field reporting and monitoring with burden on FLWs and other field staff. We believe that our HSC checklist can be streamlined and integrated into existing reporting requirements to capture key indicators without being burdensome to implement. Efforts are ongoing to digitize registers and checklists using information communication technologies for programmatic purposes in this context (Balakrishnan et al., 2016).

In conclusion, we have found that home fortification program can be delivered through FLWs, but routine monitoring is essential to track and course correct when utilizing existing government resources. In addition, our study provides evidence for the utility of PIPs and assessment of both supply and demand side factors to gain a comprehensive picture of program implementation.

## CONFLICTS OF INTEREST

The authors declare that they have no conflicts of interest.

## CONTRIBUTIONS

RM (Mehta) was responsible for overall management of the project and data collection, analysis, and drafting of this manuscript. MY oversaw the implementation of this pilot. IC and RM (Mehta) oversaw field implementation and provided technical inputs to the midline for this pilot. MY, AWG, UR, IC, PV, PK, SS, and RM were involved in design of the monitoring strategy for this pilot. MY, AWG, PK, RM, and UR provided critical insights on the manuscript. SS and RM were responsible for procuring funds for this study. All authors have reviewed and approved the final draft of this manuscript.

## Supporting information

Data S1. Supporting information

Table S1: Midline HH Survey Summary
Table S2: Midline FLW Survey Summary
Table S3: Health Sub‐Centre Checklist – Intervention
