# Supplementary material for: Use of monitoring data to improve implementation of a home fortification program in Bihar, India
Source: Matern Child Nutr. 2018 Dec 13;15(3):e12753. doi: 10.1111/mcn.12753 (PMC6617994; doi:10.1111/mcn.12753)
Supplement: Supplementary file 1 — Data S1. Supporting information [file MCN-15-e12753-s001.pdf]

Supplementary Figures 1-2: MNP intervention group informational brochure

इस पाउडर के क्या फायदे हैं?

अक्सर हमारे रोज के खाने में कई सूक्ष्म तत्वों की कमी पाई जाती है,जो खून बनाने के लिए जरूरी हैं, जिनकी कमी के कारण सामान्यतः बच्चे खून की कमी के शिकार बनते हैं। इस पाउडर से बच्चों में खून की कमी से बचाव हो सकता है। इसके अन्य संभव फायदे निम्न हैं :

- ★ बच्चे के शारीरिक व बौधिक विकास में सुधार
- ★ बच्चे की रोगों से लड़ने की क्षमता बढ़ाना
- ★ बच्चे की भूख बढ़ाना
- ★ बच्चे की सक्रियता व चंचलता को बढ़ाना

यह पाउडर कोई दवा नहीं है जो बच्चे के वर्तमान के बिमारियों को दूर कर सके। छोटी उम्र में बच्चों को यह पाउडर देने से उसके भविष्य के स्वास्थ्य की नींव मजबूत बन सकती है।

याद रखें

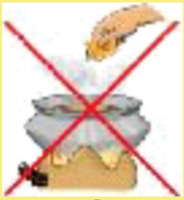

पाउडर को खाना पकाते समय या अधिक गरम खाने में नहीं मिलाएँ। ऐसा करने से खाने का स्वाद बदल सकता है।

पाउडर को चावल जैसे ठोस खाने में मिलाकर खिलाना ज्यादा फायदेमंद है। पानी या अन्य तरल चीजों में मिला कर ना दें।

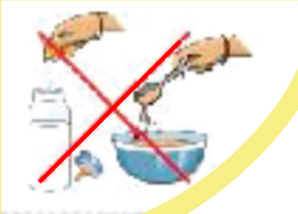

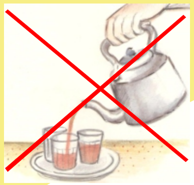

अपने बच्चे को चाय नहीं पिलाएँ। ये बच्चों में पाउडर से होने वाले फायदे को कम करता है।

यह पाउडर विशेष रुप से 6 माह से 23 माह के बच्चों के लिए बनाया गया है।

सूचना :

शुरु के कुछ दिनों में कुछ बच्चों का पैखाना काला हो सकता है। किसी-किसी बच्चों को कब्ज भी हो सकता है। इसमें घबराने की कोई बात नहीं। यदि इसके इस्तेमाल के दौरान, बच्चे को दस्त या पतला पैखाना होता है, तो यह समझें कि ऐसा किसी और कारण से हो सकता है। तीन दिन से अधिक दस्त होने पर आशा/आंगनवाड़ी दीदी या डॉक्टर से संपर्क करें। पाउडर खिलाना बंद नहीं करें। पुड़िया को साफ और सुरक्षित स्थान पर रखें।

दस्त या उल्टी होने पर बच्चे को एक पुड़िया दिन भर में तीन भाग में बाँट कर खिलाएँ

बच्चों के पोषण की सूचना

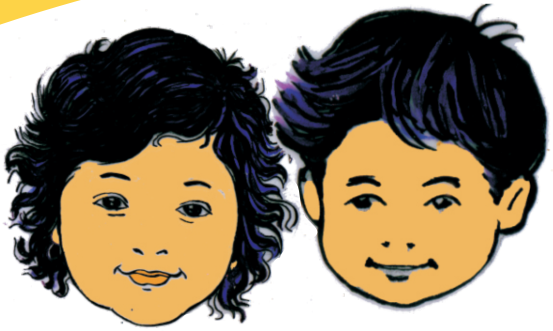

बिहार सरकार

NATIONAL HEALTH MISSION
राष्ट्रीय स्वास्थ्य मिशन

care

ananya
A partnership supported by the Bill & Melinda Gates Foundation

अधिक जानकारी के लिए आपके आशा/आंगनवाड़ी से सम्पर्क करें या दिए गए नम्बर पर फोन करें :

9771456246

बच्चों के पोषण की जानकारी

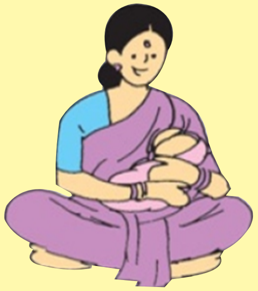

छः महीने तक बच्चे को केवल स्तनपान कराएँ। 6 माह तक माँ का दूध बच्चे की हर जरुरत को पूरा करता है; और अन्य चीज की जरूरत नहीं।

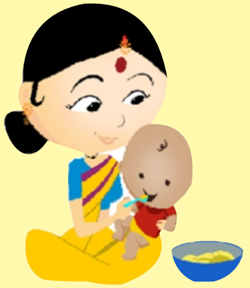

छः महीने के बाद बच्चे को स्तनपान के साथ पूरक आहार दें। साथ ही साथ स्तनपान 2 साल तक जारी रखें।

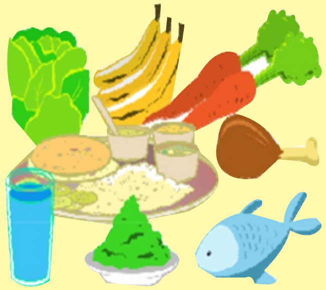

बच्चे के खाने के लिए घर में उपलब्ध सभी तरह की वस्तुओं का इस्तेमाल करें।

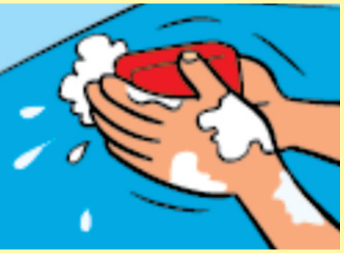

बच्चे का भोजन बनाने और खिलाने से पहले साफ सफाई पर ध्यान दें। माँ और बच्चे,दोनों का हाथ धोना याद रखें।

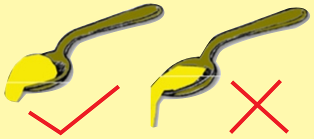

बच्चों का आहार अर्ध ठोस और गाढ़ा होना चाहिए। पतले या पानी जैसे तरल खाने से पूरा पोषण नहीं मिलता।

बच्चे के लिए एक अलग कटोरी रखें और उससे खिलाएँ। कटोरी का माप जानने के लिए आपके आशा या आंगनवाड़ी से सम्पर्क करें।

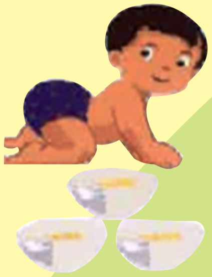

6 से 8 माह
एक कटोरी से थोड़ा कम दिन भर में 3 बार खिलाएँ।

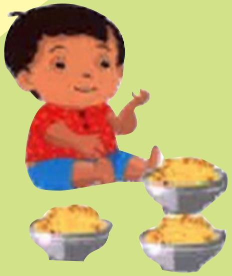

9 से 11 माह
दिन भर में 3 कटोरी और बीच में कुछ और खिलाएँ।

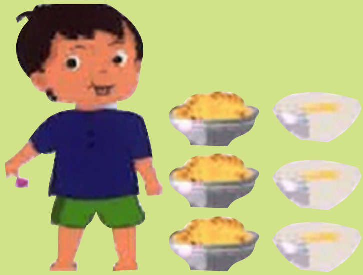

1 से 2 साल
दिन भर में डेढ़ कटोरी 3 बार और साथ में कुछ और खिलाएँ।

पाउडर के बारे में जानकारी

यह पुष्टिवर्धक पाउडर क्या है?

यह पाउडर कई सूक्ष्म पोषक तत्वों से बना है जो बच्चों में खून की कमी जैसी गंभीर समस्या से बचाव करते हैं। बच्चों को उनके सामान्य विकास और वृद्धि के लिए प्रतिदिन जितनी मात्रा में इन तत्वों की जरुरत है, उतनी मात्रा में निम्न तत्व हर एक पुड़िया में डाले गए हैं:

- ★ आयरन (लौह तत्व)
- ★ विटामिन–सी
- ★ फोलिक एसिड
- ★ आयोडिन
- ★ विटामिन–ए
- ★ विटामिन–बी 12
- ★ जिंक

यह पाउडर खाने के साथ मिलाकर खिलाया जाता है, मगर इससे खाने का स्वाद या रंग नहीं बदलता है।

हर दिन एक पाकिट पाउडर बच्चे के एक समय के खाने में मिलाएँ। एक दिन में एक से अधिक पुड़िया देने की कोई जरुरत नहीं है।

** केवल सूखे चावल या भात में पाउडर न डालें, हमेशा चावल/भात में दाल, सब्जी के साथ पाउडर मिलाकर खिलाएँ।

इस्तेमाल करने का तरीका क्या है?

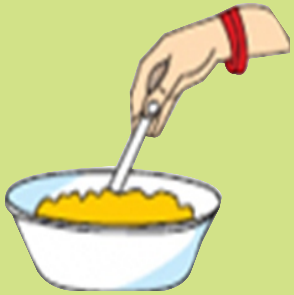

1. बच्चे का खाना पहले अलग कटोरी या थाली में तैयार कर लें।

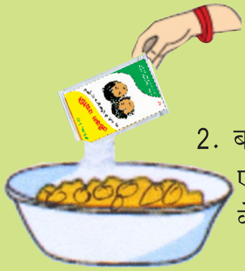

2. बच्चे को खाना खिलाने से पहले, एक पुड़िया का पाउडर बच्चे के खाने में अच्छे से मिलाएँ।

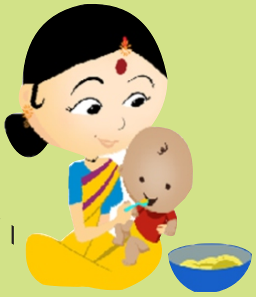

3. पाउडर मिलाया हुआ खाना बच्चे को एक घंटे के अंदर खिलाएँ।

एक माह के अंतराल में बच्चे को केवल एक डब्बा या 30 पुड़िया खिलानी है

Supplementary Figures 2-4: MNP control group informational brochure

बच्चे के लिए एक अलग कटोरी रखें और उससे खिलाएँ। कटोरी का माप जानने के लिए आपके आशा या आंगनवाड़ी से सम्पर्क करें।

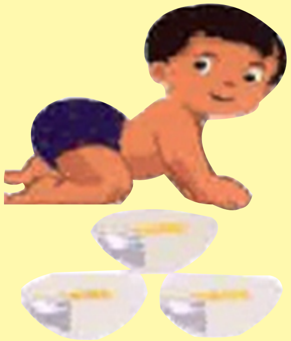

6 से 8 माह

एक कटोरी से थोड़ा कम दिन भर में 3 बार खिलाएँ।

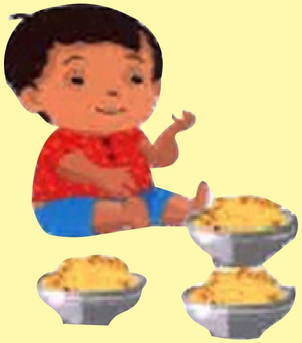

9 से 11 माह

दिन भर में 3 कटोरी और बीच में कुछ और (जैसेः फल, अंडा आदि) खिलाएँ।

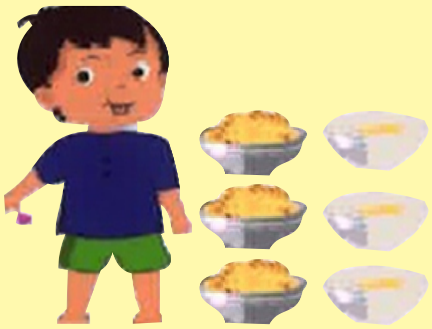

1 से 2 साल

दिन भर में डेढ़ कटोरी 3 बार और साथ में कुछ और (जैसेः फल, अंडा आदि) खिलाएँ।

बच्चों के पोषण की सूचना

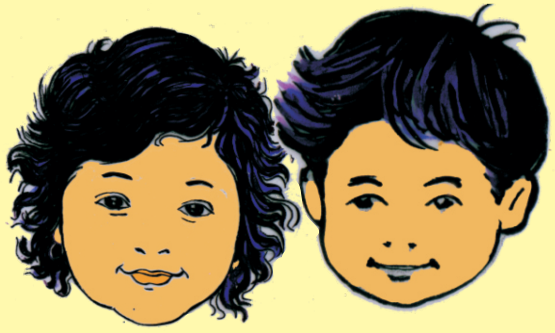

बिहार सरकार

NATIONAL HEALTH MISSION
राष्ट्रीय स्वास्थ्य मिशन

care®

ananya
A partnership supported by the Bill & Melinda Gates Foundation

अधिक जानकारी के लिए आपके आशा/आंगनवाड़ी से सम्पर्क करें या दिए गए नम्बर पर फोन करें :

9771456246

बच्चों के पोषण की जानकारी

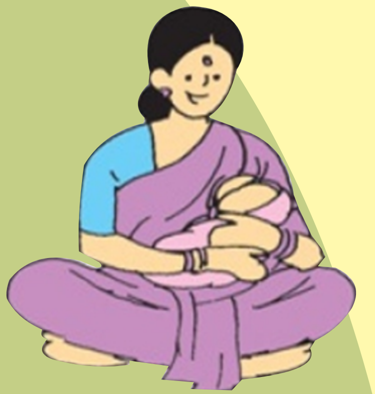

छः महीने तक बच्चे को केवल स्तनपान कराएँ। 6 माह तक माँ का दूध बच्चे की हर जरुरत को पूरा करता है; और अन्य चीज की जरूरत नहीं।

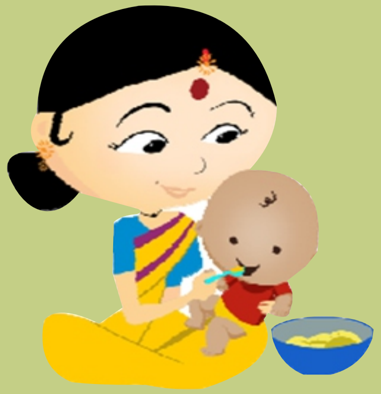

छः महीने के बाद बच्चे को स्तनपान के साथ पूरक आहार दें। साथ ही साथ स्तनपान 2 साल तक जारी रखें।

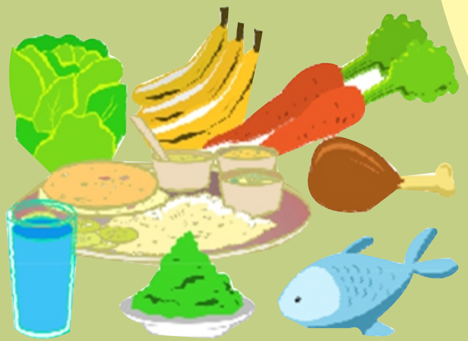

बच्चे के खाने के लिए घर में उपलब्ध सभी तरह की वस्तुओं का इस्तेमाल करें।

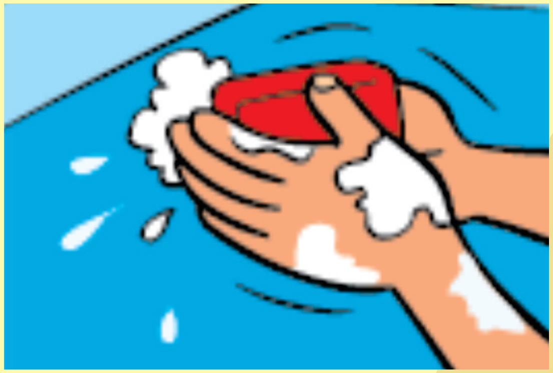

बच्चे का भोजन बनाने और खिलाने से पहले साफ सफाई पर ध्यान दें। माँ और बच्चे, दोनों का हाथ धोना याद रखें।

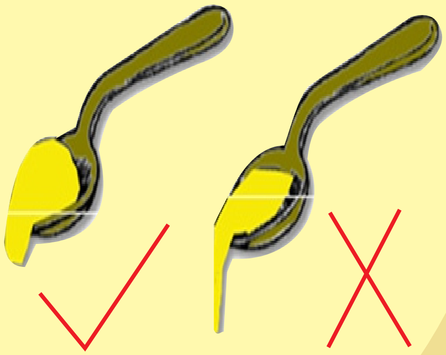

बच्चों का आहार अर्ध ठोस और गाढ़ा होना चाहिए। पतले या पानी जैसे तरल खाने से पूरा पोषण नहीं मिलता।
