# Supplementary material for: Use of monitoring data to improve implementation of a home fortification program in Bihar, India
Source: Matern Child Nutr. 2018 Dec 13;15(3):e12753. doi: 10.1111/mcn.12753 (PMC6617994; doi:10.1111/mcn.12753)
Supplement: Supplementary file 2 — Table S1: Midline HH Survey Summary Table S2: Midline FLW Survey Summary Table S3: Health Sub‐Centre Checklist – Intervention [file MCN-15-e12753-s002.docx]

**Supplementary Materials:**

| **Table 1: Midline HH Survey Summary** | Intervention | | Control | |
| --- | --- | --- | --- | --- |
|  | n | % | n | % |
| **KEY SUMMARY** | | | | |
| Sample size | 562 |  | 279 |  |
| HH Received MNP Supply in last month | 222 | 39.5 | 1 | 0.36 |
| Child consumed MNP in last week | 199 | 35.4 | 0 | 0 |
| If received MNP supply in last month, child consumed MNP in last week | 158 | 70.9 | -- | -- |
| **FLW INTERACTION** | | | | |
| Visited by AWW/ASHA in last month | 420 | 74.7 | 222 | 79.6 |
| ASHA was last FLW to visit | 283 | 67.1 | 141 | 63.5 |
| Content discussed in last visit |  |  |  |  |
| CF initiation | 247 | 44.0 | 141 | 50.5 |
| CF amount | 141 | 25.1 | 94 | 33.7 |
| CF Frequency | 96 | 17.1 | 53 | 19.0 |
| CF Diversity | 43 | 7.7 | 34 | 12.2 |
| CF Consistency | 56 | 10.0 | 45 | 16.1 |
| BF | 40 | 7.1 | 16 | 5.7 |
| Hygiene | 190 | 33.8 | 87 | 31.2 |
| MNP | 203 | 36.1 | 0 | 0.0 |
| **Program Reach and Product Use** | | | | |
| Have you heard or seen MNP? (yes) | 428 | 76.2 | 2 | 0.7 |
| FLW gave information on MNP | 411 | 96.3 | -- | -- |
| Ever received MNP | 425 | 99.3 | 0 |  |
| How do you obtain MNP? |  |  | -- | -- |
| Go to AWC | 171 | 40.3 |  |  |
| ASHA bring to home | 91 | 21.5 |  |  |
| AWW bring to home | 147 | 34.7 |  |  |
| Has child ever used MNP | 402 | 94.8 |  |  |
| Is child currently taking MNP | 174 | 43.1 |  |  |
| How often do you give your child MNP |  |  |  |  |
| > once/day | 54 | 13.6 |  |  |
| once/day | 317 | 79.9 |  |  |
| How many sachets have been used in past week for child |  |  |  |  |
| 0 | 210 | 52.2 |  |  |
| 1- 6 | 117 | 29.1 |  |  |
| 7 | 70 | 17.4 |  |  |
| >7 (8, 10,12,14,30) | 5 | 1.2 |  |  |
| Are other HH consuming product (no) | 387 | 96.3 |  |  |
| Do you intend to use product in future for child (yes) | 388 | 91.4 |  |  |
| **Program Reach and Product Use** | | | | |
| Reasons why not using or stopped using product |  |  |  |  |
| Forgot to give MNP | 19 | 3.4 |  |  |
| Child experienced side effects | 33 | 5.9 |  |  |
| Child does not like food when MNP added | 26 | 4.6 |  |  |
| Child has poor appetite/does not eat | 19 | 3.4 |  |  |
| Child not eating food yet | 9 | 1.6 |  |  |
| Child has been sick | 17 | 3.0 |  |  |
| No supply | 118 | 21.0 |  |  |
| What type of food is MNP mixed into |  |  |  |  |
| semi-solid | 350 | 86.4 |  |  |
| solid | 46 | 11.4 |  |  |
| water/thin food/liquids (milk, juice) | 9 | 2.2 |  |  |
| MNP added after cooking and before serving to child | 399 | 99.8 |  |  |

| **Table 2: Midline FLW Survey Summary** | Intervention | | Control | |
| --- | --- | --- | --- | --- |
|  | n | %  Mean/SD/Range | n | %  Mean/SD/Range |
| **HH VISITS and OTHER PROGRAMS** | | | | |
| Sample size | 278 |  | 140 |  |
| Participated in an HSC meeting this year | 277 | 99% | 140 | 100% |
| Participate in HSC meeting every month or every time there is a meeting | 219 | 79% | 116 | 83% |
| Discussed IYCF in HSC meeting | 277 | 100% | 139 | 100% |
| **FLW KNOWLEDGE- HIGH** | | | | |
| For how many months should a child receive only breastmilk? | 278 | 6 ± 1 months | 140 | 6 ± 0.2 months |
| When should infant first start to receive semi-solid food? | 277 | 6.9 ± 0.4 months | 139 | 6.8 ± 0.4 months |
| How long should children continue to receive breastmilk along with complementary food? | 265 | 25 ± 6 months | 136 | 24 ± 6 months |
| At minimum, how many times a day should a 4 month old be fed semi-solid food? | 184 | 0 ± .2 meals /day | 105 | 0 ± 0 meals /day |
| At minimum, how many times a day should a 6-8 month old be fed semi-solid food? | 277 | 2.8 ± .6 meals /day | 139 | 3.2 ± 2.5 meals /day |
| At minimum, how many times a day should a 12-23 month old be fed semi-solid food? | 277 | 4.2 ± 1 meals /day | 139 | 5.1 ± 6 meals /day |
| **PROGRAM IMPLEMENTATION** | | | | |
| Have you received correct pamphlet on child feeding? (yes) | 247 | 89% | 113 | 81% |
| I always have adequate supply of pamphlets | 139 | 55% | 78 | 65% |
| Have you ever heard of MNP? | 275 | 99% | 101 | 72% |
| Have your HSC meetings included information on MNP? | 270 | 99% | 27 | 26% |
| Have you received MNP to distribute to HHs? | 254 | 93% | 2 | 2% |
| How frequently do you receive MNP? (only once) | 250 | 96% | 1 | --- |
| Have you ever ran out of MNP? (yes) | 84 | 32% | 1 | --- |
| Do you have a supply of MNP right now? (yes) | 213 | 82% | 1 | --- |
| Have you ever given MNP to HH? (yes) | 253 | 99% | 1 | --- |
| Most often how do you distribute (home visits) | 244 | 93% | 1 | --- |
| How often do you visit a HH with child 6-17 mo? (1/mo) | 201 | 77% | 1 | --- |
| Have any children experienced side effects (yes) | 60 | 23% | 0 | --- |
|  | | | | |
|  | Intervention | | Control | |
|  | n | %  Mean/SD | n | %  Mean/SD |
| **FLW OPINIONS** | | | | |
| I think counseling is an important part of my role (agree) | 278 | 100% | 140 | 100% |
| I think right nutrition of the child is very important for the child’s health and development (agree) | 277 | 100% | 140 | 100% |
| I think the HSC trainings have helped improve my knowledge of child feeding and nutrition (agree) | 278 | 100% | 139 | 99% |
| I think it’s important to provide MNP to young children in my community (agree) (*only asked of FLWs who have distributed the powder-Note may need to retrain on enumeration skip patterns and look into why responses are so high in control?*) | 272 | 98% | -- | -- |
| I think that the MNP program has added to my workload (agree) | 137 | 49% | -- | -- |
| I think the MNP program has improved my status in the community (agree) | 264 | 96% | -- | -- |
| I want to continue to distribute MNP in the future in my community (agree) | 264 | 96% | -- | -- |

| **Table 3: Health Sub-Centre Checklist – Intervention** | | | | | | | | | | | | | | | | | | | |
| --- | --- | --- | --- | --- | --- | --- | --- | --- | --- | --- | --- | --- | --- | --- | --- | --- | --- | --- | --- |
| **Block:** | | | | | |  | | | | | | | | | | | | | |
| **Name of HSC:** | | | | | |  | | | | | | | | | | | | | |
| **Date of HSC Meeting:** | | | | | |  | | | | | | | | | | | | | |
| **Name of Person Completing Checklist:** | | | | | |  | | | | | | | | | | | | | |
| **Position of Person Completing Checklist:** | | | | | |  | | | | | | | | | | | | | |
| **Total # of FLWs at Meeting:** | | | | | |  | | | | | | | | | | | | | |
| **Code for FLWs:** | | | | | |  | | | | | | | | | | | | | |
| **Please complete the questions listed below. Responses to these questions will not be used to judge your performance. This data will help us improve this program and make it successful. Please complete this information as correctly as possible.** | | | | | | | | | | | | | | | | | | | |
| 1. **Who conducted this HSC meeting?** | | | | | | ANM: ______________________________________________________  HSC Facilitator: _______________________________________________  Other: ______________________________________________________ | | | | | | | | | | | | | |
| 1. **Total FLWs who participated in this meeting**   Attn: This information should pertain to FLWs who belong to this HSC. For FLWs from other HSCs who may have attended this meeting, please enter their information in “other” | | | | | | ASHAs: __ __  AWC: __ __ __ __ __ __ __ __ __ __ __ __ __ __ __ __ __ __ __ __ __ __ __ __  __ __ __ __ __ __ __ __ __ __ __ __ __ __ __ __ __ __ __ __ __ __ __ __  AWWs: __ __  AWC: __ __ __ __ __ __ __ __ __ __ __ __ __ __ __ __ __ __ __ __ __ __ __ __  __ __ __ __ __ __ __ __ __ __ __ __ __ __ __ __ __ __ __ __ __ __ __ __  Other: __ __  AWC: __ __ __ __ __ __ __ __ __ __ __ __ __ __ __ __ __ __ __ __ __ __ __ __  __ __ __ __ __ __ __ __ __ __ __ __ __ __ __ __ __ __ __ __ __ __ __ __ | | | | | | | | | | | | | |
| 1. **Total FLWs who were absent from this meeting** | | | | | | ASHAs: __ __  AWC: __ __ __ __ __ __ __ __ __ __ __ __ __ __ __ __ __ __ __ __ __ __ __ __  __ __ __ __ __ __ __ __ __ __ __ __ __ __ __ __ __ __ __ __ __ __ __ __  AWWs: __ __  AWC: __ __ __ __ __ __ __ __ __ __ __ __ __ __ __ __ __ __ __ __ __ __ __ __  __ __ __ __ __ __ __ __ __ __ __ __ __ __ __ __ __ __ __ __ __ __ __ __  Other: __ __  AWC: __ __ __ __ __ __ __ __ __ __ __ __ __ __ __ __ __ __ __ __ __ __ __ __  __ __ __ __ __ __ __ __ __ __ __ __ __ __ __ __ __ __ __ __ __ __ __ __ | | | | | | | | | | | | | |
| 1. **Was information about breastfeeding provided in this meeting?** | | | | | | YES: __________________  NO: __________________ | | | | | | | | | | | | | |
| 1. **Was IYCF information discussed during this meeting?** | | | | | | YES: __________________  NO: __________________ | | | | | | | | | | | | | |
| 1. **Was any IYCF related demonstration using a katori and spoon conducted during this meeting to show how a child should be given complementary foods?** | | | | | | YES: __________________  NO: __________________ | | | | | | | | | | | | | |
| 1. **Has any information been provided/discussion had about Jeevan Jyoti (MNP) power?** | | | | | | YES: __________________  NO: __________________ | | | | | | | | | | | | | |
| 1. **For the last month, please complete information about IYCF pamphlets and MNP Stock**   Note: For each column, please enter 1 or 2. If an AWC reports a lack of supplies, please immediately contact CARE staff after this meeting. | | | | | | | | | | | | | | | | | | | |
| **AWC Code** | | **MNP Stocks and Supplies**  **(Enter 1 or 2 as indicated below)** | | | | | | **To date, how many boxes of MNP have you distributed?** | | **Have adequate stocks of pamphlets**  (1 – Yes \| 2 – No \| 3 – don’t know) | | **# of HH visited to counsel on IYCF and Complementary Feeding** | | **# of HH where MNP and pamphlets were distributed** | | | **Comments** | | |
|  |  | Have received supplies  (1 – received \|  2- not received \|  3 – don’t know) | | Have adequate supplies  (1 – Yes \| 2 – No \| 3 – don’t know) | | | |  |  |  |  |  |  |  |  |  |  |  |  |
|  | |  | |  | | | |  | |  | |  | |  | | |  | | |
|  | |  | |  | | | |  | |  | |  | |  | | |  | | |
|  | |  | |  | | | |  | |  | |  | |  | | |  | | |
|  | |  | |  | | | |  | |  | |  | |  | | |  | | |
|  | |  | |  | | | |  | |  | |  | |  | | |  | | |
| 1. **Information about morbidity and mortality in catchment: Please complete the section below based on information provided by ASHAs and AWWs. If there is no morbidity or mortality to report for a particular catchment in the last month, please write “0” for that AWC.**   Note: If there is no information for an AWC, please write “99”. | | | | | | | | | | | | | | | | | | | |
|  | **Maternal Mortality:**  Maternal deaths which have occurred during 9 months of pregnancy or within 42 days after child birth | | | | | | **Infant and Young Child Mortality:**  Death of a child between 0-18 months | | | | | | **Child Morbidity:**  Illness among children 6-18 months | | | | | | |
| **AWC Code** | **# of Deaths**  (99 if don’t know) | | **Reason for Death** | | **Age of Woman**  **(in Yrs)** | | **# of Deaths**  (99 if don’t know) | | **Reason for Death** | | **Age of Child**  **(in Days or Months)** | | **Total Cases of Diarrhea** | | | **Total Cases of Pneumonia** | | | **Total Cases of Vomiting** |
|  |  |  |  |  |  |  |  |  |  |  |  |  | 99 if don’t know | | | | | | |
|  |  | |  | |  | |  | |  | |  | |  | |  | | |  | |
|  |  | |  | |  | |  | |  | |  | |  | |  | | |  | |
|  |  | |  | |  | |  | |  | |  | |  | |  | | |  | |
|  |  | |  | |  | |  | |  | |  | |  | |  | | |  | |
| In case of any difficulties or important and immediate questions, please contact the Innovation Coordinator at: ___________________________________ | | | | | | | | | | | | | | | | | | | |
